# The triglyceride-glucose index as an independent associate of severe tubulointerstitial fibrosis and a predictor of renal survival in IgA nephropathy

**DOI:** 10.3389/fendo.2026.1754033

**Published:** 2026-01-23

**Authors:** Guijing Tang, Anni Wang, Xingyu Zhu, Danyan Yu, Hongyu Chen, Xue Jiang, Hong Liu

**Affiliations:** 1 Department of Nephrology, Hangzhou Traditional Chinese Medicine (TCM) Hospital Affiliated to Zhejiang Chinese Medical University, Hangzhou, Zhejiang, China; 2 Key Laboratory of Precise Prevention and Treatment of Rheumatism Syndrome of Renal Wind Disease, Hangzhou, Zhejiang, China

**Keywords:** IgA nephropathy, insulin resistance, prognosis, renal survival, triglyceride-glucose (TyG) index, tubular atrophy/interstitial fibrosis

## Abstract

**Background:**

To investigate the association of the triglyceride-glucose (TyG) index with severe tubular atrophy/interstitial fibrosis (T2 lesions) and renal outcomes in patients with IgA nephropathy (IgAN).

**Methods:**

We retrospectively analyzed 1,791 biopsy-proven IgAN patients between October 2014 and September 2024. Receiver operating characteristic (ROC) curve analysis assessed TyG index performance for predicting T2 lesions. Multivariable logistic regression and restricted cubic spline (RCS) models were applied to assess the relationship between the TyG index and T2 lesions. Renal survival was evaluated using Kaplan-Meier analysis and Multivariable Cox regression analysis.

**Results:**

Among 1,791 enrolled IgAN patients, 122 (6.8%) exhibited T2 lesions. The TyG index showed predictive value for T2 lesions (AUC = 0.699, 95%CI: 0.656-0.741), with an optimal cut-off of 8.69 (specificity 64.5%, sensitivity 65.6%). After 1:1 PSM, the high-TyG group (>8.69) had a higher prevalence of severe tubulointerstitial lesions, elevated BMI, serum creatinine, 24-hour urinary protein, lower eGFR (all P < 0.001), and a higher incidence of the primary endpoint (P = 0.020). Multivariable logistic regression confirmed that a high TyG index (TyG>8.69) was independently associated with T2 lesions (OR = 2.365, 95% CI: 1.104-5.062, P = 0.027). RCS analysis indicated a linear dose-response relationship (P for overall<0.001;P for nonlinearity=0.082). Kaplan-Meier analysis showed significantly worse renal survival in the high-TyG group both before and after matching (log-rank=13.59, P<0.001 after matching). Multivariable Cox regression identified a high TyG index as an independent predictor of adverse renal outcomes (HR = 1.561, 95% CI: 1.046–2.330, P = 0.029).

**Conclusion:**

The TyG index is independently associated with severe tubulointerstitial damage and poor renal survival in IgAN, with a value >8.69 effectively identifying high-risk patients for progressive renal injury.

## 1 Introduction

IgA nephropathy (IgAN) is the most prevalent primary glomerulonephritis worldwide and a leading cause of end-stage renal disease (ESRD). It is estimated that 25%–50% of patients progress to ESRD within 20–30 years after diagnosis (1, 2). Pathologically, IgAN is characterized by dominant mesangial immunoglobulin A deposition on renal biopsy (3). Among the histologic criteria outlined in the Oxford Classification, tubular atrophy/interstitial fibrosis has been consistently validated as the strongest predictor of adverse renal outcomes (4, 5). However, the assessment of these tubulointerstitial lesions currently relies exclusively on renal biopsy—an invasive procedure with inherent sampling variability and clinical limitations. Consequently, there is an urgent need to identify and develop non-invasive biomarkers capable of evaluating tubulointerstitial injury and enhancing risk stratification in patients with IgAN.

The triglyceride-glucose (TyG) index, derived from fasting triglyceride and glucose levels, has been widely adopted as a reliable surrogate marker for insulin resistance (IR) (6). Accumulating evidence supports its clinical relevance across a spectrum of metabolic and cardiovascular diseases, including metabolic syndrome (7), type 2 diabetes (8), ischemic stroke (9), heart failure (10), hypertension (11), and chronic kidney disease (12, 13). In the context of IgAN, the pioneering study by Qin A et al. (14) demonstrated that an elevated TyG index correlates with an increased risk of renal disease progression, highlighting its prognostic significance. However, the specific relationship between the TyG index and the severity of tubular atrophy/interstitial fibrosis—a critical histopathological determinant of prognosis in IgAN, remains inadequately understood. Consequently, this study aimed to investigate, for the first time, the independent association between the TyG index and severe tubulointerstitial damage (T2 lesions) and to evaluate its impact on long-term renal outcomes in a large cohort of patients with IgAN.

## 2 Materials and methods

### 2.1 Study population

**Figure 1:**
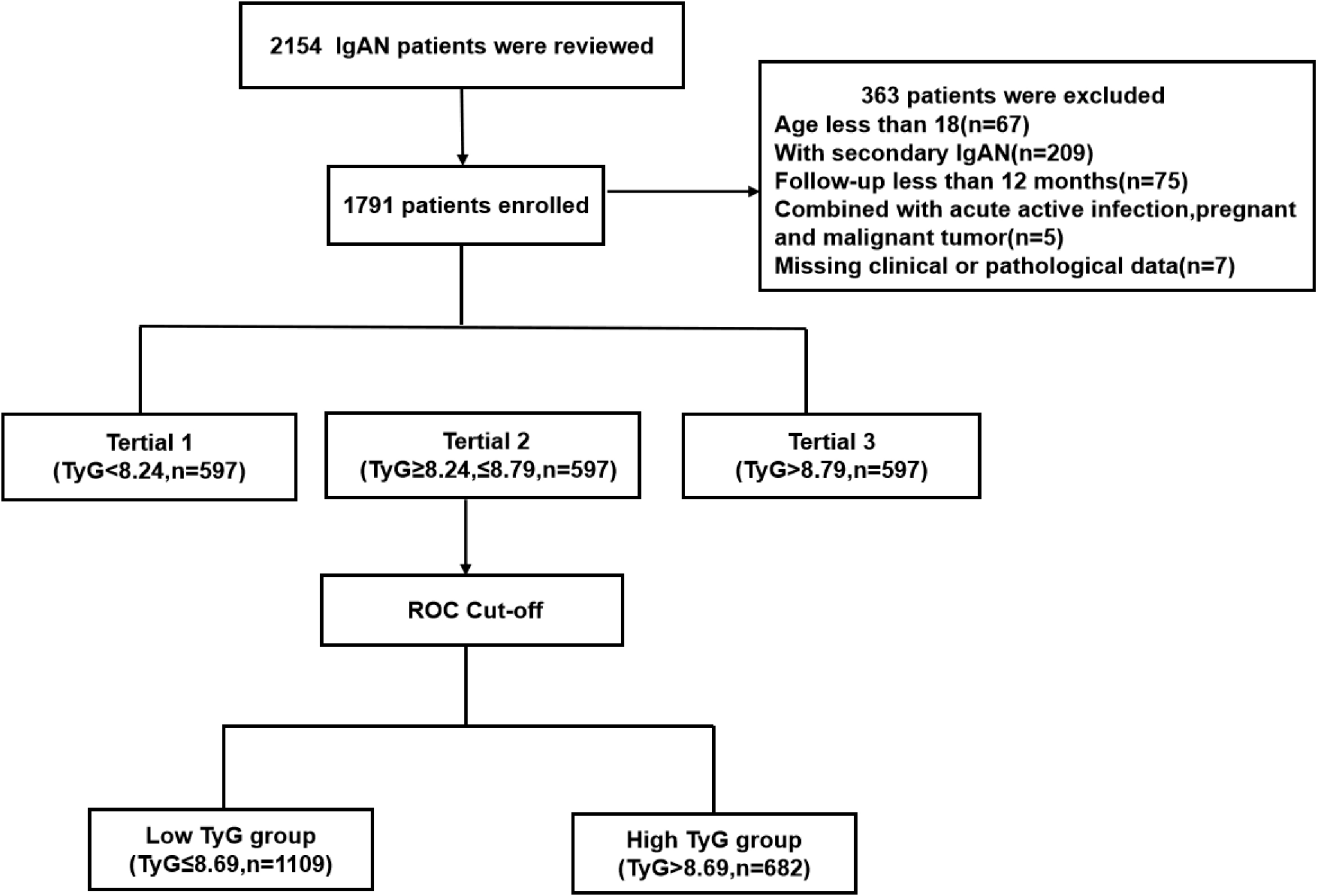
Flowchart of patient inclusion and exclusion in this retrospective cohort study. A total of 2,154 biopsy-proven IgA nephropathy patients were initially screened. After applying exclusion criteria (age <18 years, secondary IgAN, follow-up <12 months, acute comorbidities, and missing data), 1,791 patients were included in the final analysis. The flowchart illustrates the stepwise exclusion process and the final cohort composition.

This single-center retrospective cohort study initially enrolled 2,154 patients with biopsy-proven IgAN from Hangzhou Hospital of Traditional Chinese Medicine between October 2014 and September 2023. Exclusion criteria were as follows: 1) age <18 years (n=67); 2) secondary IgAN, including cases associated with diabetes mellitus, Henoch–Schönlein purpura, or autoimmune diseases (n=209); 3) follow-up <12 months (n=75); 4) acute comorbidities such as active infections, pregnancy, or malignancy (n=5); and 5) missing clinical or pathological data (n=7). After exclusions, 1,791 patients were included in the final analysis (**Figure 1**). The study protocol was approved by the Research Ethics Committee of Hangzhou Hospital of Traditional Chinese Medicine (Approval No.2024KLL230). The committee waived the requirement for informed consent due to the use of anonymized retrospective clinical data.

### 2.2 Clinical data

Baseline clinical data were collected from electronic medical records at the time of renal biopsy. Demographic characteristics (age, sex, and BMI), medical history (hypertension and smoking history), and laboratory parameters—including hemoglobin (Hb), albumin (ALB), total cholesterol (TC), triglycerides (TG), low-density lipoprotein (LDL), high-density lipoprotein (HDL), fasting blood glucose (FBG), Serum immunoglobulin A (Serum IgA), Serum immunoglobulin G (Serum IgG), Serum immunoglobulin M (Serum IgM), Serum C3, Serum C4, uric acid (UA), serum creatinine (Scr), 24-hour urinary protein excretion (24h Upro), and estimated glomerular filtration rate (eGFR)—were systematically documented. All the laboratory parameters were measured in the central laboratory of our hospital using standardized automated analyzers (Beckman Coulter AU5800, USA) according to manufacturer protocols and established internal quality control procedures. All analyses were conducted on fasting blood samples collected within one week prior to renal biopsy. Treatment regimens at biopsy were also recorded. Renal tissue specimens were evaluated by pathologists in the nephropathy laboratory at Hangzhou Hospital of Traditional Chinese Medicine according to the Oxford Classification (MEST-C) for IgAN, which includes mesangial hypercellularity (M0/M1), endocapillary hypercellularity (E0/E1), segmental glomerulosclerosis (S0/S1), tubular atrophy/interstitial fibrosis (T0/T1/T2), and crescentic lesions (C0/C1/C2).

### 2.3 Treatment and definitions

All patients received standard supportive care based on KDIGO guidelines and tailored to their individual clinical and histopathological profiles. Treatment protocols included angiotensin-converting enzyme inhibitors or angiotensin receptor blockers (ACEI/ARB), sodium-glucose cotransporter 2 inhibitors (SGLT2i), corticosteroids, and immunosuppressive agents such as cyclophosphamide, mycophenolate mofetil, tacrolimus, or cyclosporine. The TyG index was calculated by using the following formula: Ln(TG(mg/dL)× FBG(mg/dL)/2). The eGFR was calculated using the Chronic Kidney Disease Epidemiology Collaboration (CKD-EPI) equation based on serum creatinine levels measured as described above.

### 2.4 Prognosis definition

The main renal endpoint was established as a combined event, consisting of either a 50% reduction in the eGFR or the onset of ESRD, which was characterized by an eGFR<15 mL/min/1.73 m2 or the requirement for kidney replacement therapy.

### 2.5 Statistical analysis

Continuous variables with a non-normal distribution are expressed as median and interquartile range. Group comparisons were performed using the Kruskal–Wallis test (across tertiles) or the Mann-Whitney U test (between two groups). Categorical variables are presented as numbers (%) and compared using the chi-square test or Fisher’s exact test, as appropriate. The predictive ability of the TyG index for T2 lesions was evaluated using receiver operating characteristic (ROC) curve analysis, from which the optimal cutoff value, sensitivity, and specificity were derived. Correlation analyses involved Spearman’s rank correlation for continuous variables and logistic regression for categorical variables to assess associations between the TyG index and clinicopathological parameters. Propensity score matching (PSM) was implemented in a 1:1 ratio to reduce potential confounding. Multivariable logistic regression was used to identify factors independently associated with severe tubular atrophy/interstitial fibrosis. Restricted cubic splines (RCS) were applied to visualize the nonlinear relationship between the TyG index and T2 lesions. Renal survival was analyzed using Kaplan–Meier curves, and group differences were assessed with the log-rank test. A multivariable Cox proportional hazards model was employed to examine the association between elevated TyG index and renal outcomes in IgAN patients. Additionally, a multivariable ROC analysis was performed based on logistic regression to evaluate the risk factors associated with renal prognosis in IgAN. Multicollinearity was assessed through the variance inflation factor (VIF). All variables incorporated in the final multivariable models exhibited a VIF below 2.5, signifying acceptable levels of multicollinearity and a minimal risk of estimation bias in the model coefficients. All analyses were conducted using IBM SPSS Statistics (version 27.0), with a P-value < 0.05 considered statistically significant.

## 3 Results

### 3.1 Baseline characteristics of study participants

A total of 2154 patients with IgAN were involved while 363 individuals were excluded in accordance with inclusion and exclusion criteria, and 1791 patients were finally included in this study, as shown in **Figure 1**. This cohort study comprised 760 (42.4%) males and 1031 (57.6%) females, and the mean age was 43.0(36.0-53.0) years old. The median follow-up duration for the entire cohort was 45.5 months (range: 12.0–110.0 months),and during which 130 patients reached the primary renal endpoint. According to baseline TyG levels, all patients were categorized into three tertiles: Tertile 1 (<8.24, n=597, 33.3%), Tertile 2 (8.24-8.79, n=597, 33.3%), and Tertile 3 (>8.79, n=597, 33.3%). Analysis of baseline characteristics demonstrated significant differences in most clinical indicators across the groups, with the exception of hypertension prevalence, albumin, and serum IgM levels. As TyG levels increased, patients showed progressive elevations in TC, TG, LDL, FBG, Scr, UA, and 24-hour urine protein levels, alongside a gradual decline in eGFR (P<0.001). With regard to humoral immunity, the higher TyG group demonstrated significantly increased levels of serum IgA, C3, and C4 (P<0.001), as well as reduced serum IgG levels (P = 0.002).Renal pathological evaluation using the MEST-C scoring system revealed that only the T scores differed significantly among the tertiles (P<0.001). Furthermore, higher TyG levels were associated with an increased proportion of patients experiencing primary endpoint events (P < 0.001), as detailed in **Table 1**.

**Table 1:** Baseline characteristics of 1791 igAN patients according to TyG index tertiles.

Variables	Total (n=1791)	Tertile1 (n=597) <8.24	Tertile2 (n=597) ≥8.24, ≤8.79	Tertile3 (n=597) >8.79	P value
Age,years	43.0 (36.0-53.0)	42.5 (36.0-53.0)	43.0 (35.0-53.0)	43.0 (36.0-52.0)	**<0.001**
Male, n (%)	760 (42.4)	177 (29.6)	259 (43.4)	324 (54.3)	**<0.001**
BMI, (kg/m2)	23.05 (20.85-25.35)	21.22 (19.47-23.34)	23.05 (21.18-24.93)	24.97 (22.96-27.23)	**<0.001**
Hypertension, n (%)	195 (10.9)	63 (10.6)	68 (11.4)	64 (10.7)	0.886
Smoking, n (%)	172 (9.6)	32 (5.4)	54 (9.0)	86 (14.4)	**<0.001**
HB, g/L	126 (113-139)	123 (111-133)	128 (114-139)	130 (116-144)	**<0.001**
Albumin, g/L	38.5 (35.8-41.0)	38.7 (36.3-41.3)	38.8 (35.8-41.2)	38.5 (35.2-41.0)	0.067
TC, mmol/L	4.65 (4.05-5.37)	4.29 (3.75-4.86)	4.67 (4.14-5.35)	5.05 (4.44-6.00)	**<0.001**
TG, mmol/L	1.29 (0.90-1.97)	0.78 (0.64-0.91)	1.30 (1.13-1.49)	2.37 (1.96-2.99)	**<0.001**
LDL, mmol/L	2.84 (2.34-3.42)	2.47 (2.05-2.93)	2.90 (2.46-3.39)	3.17 (2.65-3.39)	**<0.001**
FBG, mmol/L	4.67 (4.30-5.11)	4.38 (4.09-4.75)	4.67 (4.37-5.04)	5.01 (4.61-5.55)	**<0.001**
UA, umol/L	365.0 (300.0-437.0)	316.5 (260.0-383.0)	362.5 (304.0-433.0)	417.0 (356.5-488.0)	**<0.001**
Scr, umol/L	80 (62-109)	68 (57-87)	83 (62-107)	96 (73-136)	**<0.001**
24h Upro, g/L	0.91 (0.45-1.80)	0.58 (0.31-1.13)	0.85 (0.47-1.78)	1.41 (0.72-2.74)	**<0.001**
eGFR, ml/min/1.73m^2^	85.20 (61.00-106.60)	99.10 (79.30-116.20)	84.00 (62.60-104.70)	69.05 (47.00-92.85)	**<0.001**
Serum IgG, mg/dl	1080 (909-1250)	1100 (924-1260)	1090 (911-1280)	1045 (885-1220)	**0.002**
Serum IgA, mg/dl	295 (233-362)	279 (223-346)	294 (233-360)	305 (245-378)	**<0.001**
Serum IgM, mg/dl	104 (74-139)	107 (77-145)	104 (71-137)	101 (71-136)	0.107
Serum C3, mg/dl	97 (86-110)	88 (79-98)	96 (87-108)	106 (95-118)	**<0.001**
Serum C4, mg/dl	23 (19-28)	20 (17-25)	23 (19-27)	26 (22-30)	**<0.001**
Mesangial cellularity, n (%)					
M0	22 (1.3)	5 (0.8)	8 (1.3)	9 (1.5)	0.550
M1	1769 (98.7)	592 (99.2)	589 (98.7)	588 (98.5)	
Endocapillary hypercellularity, n (%)					
E0	1232 (68.8)	400 (67.0)	410 (68.7)	422 (70.7)	0.388
E1	559 (31.2)	197 (33.0)	187 (31.3)	195 (29.3)	
Segmental sclerosis, n (%)					
S0	345 (19.3)	117 (19.6)	110 (18.4)	118 (19.8)	0.815
S1	1446 (80.7)	480 (80.4)	487 (81.6)	479 (80.2)	
Tubular atrophy/interstitial fibrosis, n (%)					
T0	1255 (70.1)	499 (83.6)	421 (70.5)	335 (56.1)	**<0.001**
T1	414 (23.1)	86 (14.4)	138 (23.1)	190 (31.8)	
T2	122 (6.8)	12 (2.0)	38 (6.4)	72 (12.1)	
Crescents, n (%)					
C0	642 (35.8)	204 (34.2)	216 (36.2)	222 (37.2)	0.410
C1	1004 (56.1)	349 (58.5)	336 (56.3)	319 (53.4)	
C2	145 (8.1)	44 (7.3)	45 (7.5)	56 (9.4)	
Steroid with/without immunosuppressants treatment, n (%)	512 (28.6)	162 (27.1)	172 (28.8)	168 (28.1)	0.585
Using lipid-lowering drugs, n (%)	354 (19.8)	119 (19.9)	109 (18.3)	126 (21.1)	0.463
Using SGLT2i drugs, n (%)	383 (21.4)	125 (20.9)	129 (21.6)	129 (21.7)	0.948
Using ACEI/ARB drugs, n (%)	1441 (80.5)	481 (80.5)	482 (80.7)	478 (80.0)	0.955
Composite event, n (%)	130 (7.3)	20 (3.4)	41 (6.9)	69 (11.6)	**<0.001**

Continuous variables are expressed as median (interquartile range); Categorical variables are expressed as frequency (%); Bold values was that the differences were significant (P<0.05).

BMI, body mass index; HB, hemoglobin; TC, total cholesterol; TG, triglyceride; LDL, low-density lipoprotein; FBG, fasting blood glucose; UA, uric acid; Scr, serum creatinine; 24h Upro:24-hour urine protein; eGFR, estimated glomerular filtration rate; SGLT2i, sodium-glucose cotransporter-2 inhibitors; ACEI/ARB, angiotensin-converting enzyme inhibitors/angiotensin II receptor blockers.

### 3.2 Correlation of the TyG index with clinical parameters and pathological lesions

Correlation analysis was performed to evaluate the relationships between the TyG index and key clinicopathological parameters. As summarized in **Table 2**, the TyG index exhibited significant positive correlations with male sex (r = 0.223, P < 0.001), age (r = 0.308, P < 0.001), BMI (r = 0.465, P < 0.001), smoking status (r = 0.150, P < 0.001), Hb(r = 0.187, P < 0.001), UA(r = 0.398, P < 0.001), Scr(r = 0.340, P < 0.001),24-hour urine protein (r = 0.368, P < 0.001),Serum IgA(r = 0.109, P < 0.001),Serum C3(r = 0.437, P < 0.001) and Serum C4(r = 0.327, P < 0.001). Conversely, it was negatively correlated with ALB(r= -0.073, P = 0.002),eGFR(r= -0.356, P < 0.001) and Serum IgG(r = -0.125, P < 0.001). Correlation analysis between the TyG index and renal pathological parameters indicated that tubular atrophy/interstitial fibrosis(T) was the only factor exhibiting a statistically significant positive correlation with the TyG index (r = 0.257, P < 0.001).

**Table 2:** Correlation analysis of the TyG index with clinicopathological characteristics.

	Variables	Correlation coefficient(r)	P value
TyG	sex	0.223	**<0.001**
	age	0.308	**<0.001**
	BMI	0.465	**<0.001**
	Smoking	0.150	**<0.001**
	Hypertension	0.009	0.701
	Hb	0.187	**<0.001**
	ALB	-0.073	**0.002**
	UA	0.398	**<0.001**
	Scr	0.340	**<0.001**
	24h Upro	0.368	**<0.001**
	eGFR	-0.356	**<0.001**
	Serum IgG	-0.125	**<0.001**
	Serum IgA	0.109	**<0.001**
	Serum C3	0.437	**<0.001**
	Serum C4	0.327	**<0.001**
	M	-0.037	0.118
	E	-0.025	0.288
	S	-0.012	0.624
	T	0.257	**<0.001**
	C	-0.005	0.846

Bold values was that the differences were significant (P<0.05).

TyG, the triglyceride-glucose (TyG) index; BMI, body mass index; Hb, hemoglobin; ALB,albumin; UA, uric acid; Scr, serum creatinine; 24h Upro, 24-hour urine protein; eGFR, estimated glomerular filtration rate; M, mesangial hypercellularity; E,endocapillary hypercellularity; S, segmental glomerulosclerosis; T, tubular atrophy/interstitial fibrosis; C, crescentic lesions.

### 3.3 Predictive value of the TyG index for severe tubular atrophy/interstitial fibrosis

**Figure 2:**
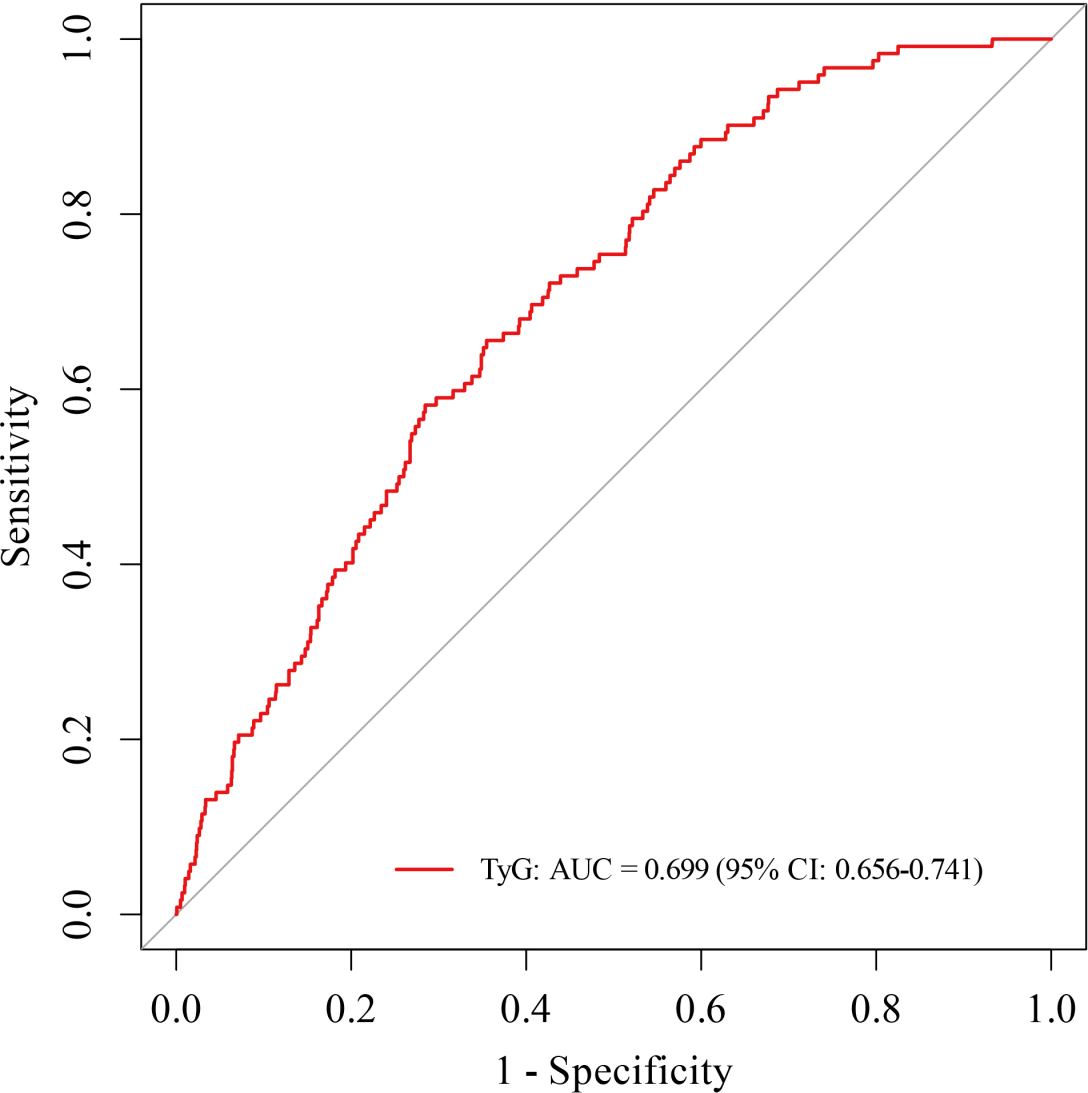
Receiver operating characteristic (ROC) curve of the TyG index for predicting severe tubular atrophy/interstitial fibrosis (T2 lesions) in IgAN. The area under the curve (AUC) was 0.699 (95% CI: 0.656–0.741). The optimal cutoff value for the TyG index was 8.69, with a sensitivity of 65.6% and a specificity of 64.5%. AUC, the area under the receiver operating characteristic curve.

The predictive performance of the TyG index for identifying severe tubular atrophy and interstitial fibrosis (T2 lesions) in patients with IgAN was evaluated using ROC curve analysis. The AUC was 0.699 (95% CI: 0.656–0.741). An optimal TyG index cut-off value of 8.69 was determined, corresponding to a sensitivity of 65.6% and a specificity of 64.5% (**Figure 2**).

### 3.4 Nonlinear association between the TyG index and severe tubular atrophy/interstitial fibrosis

**Figure 3:**
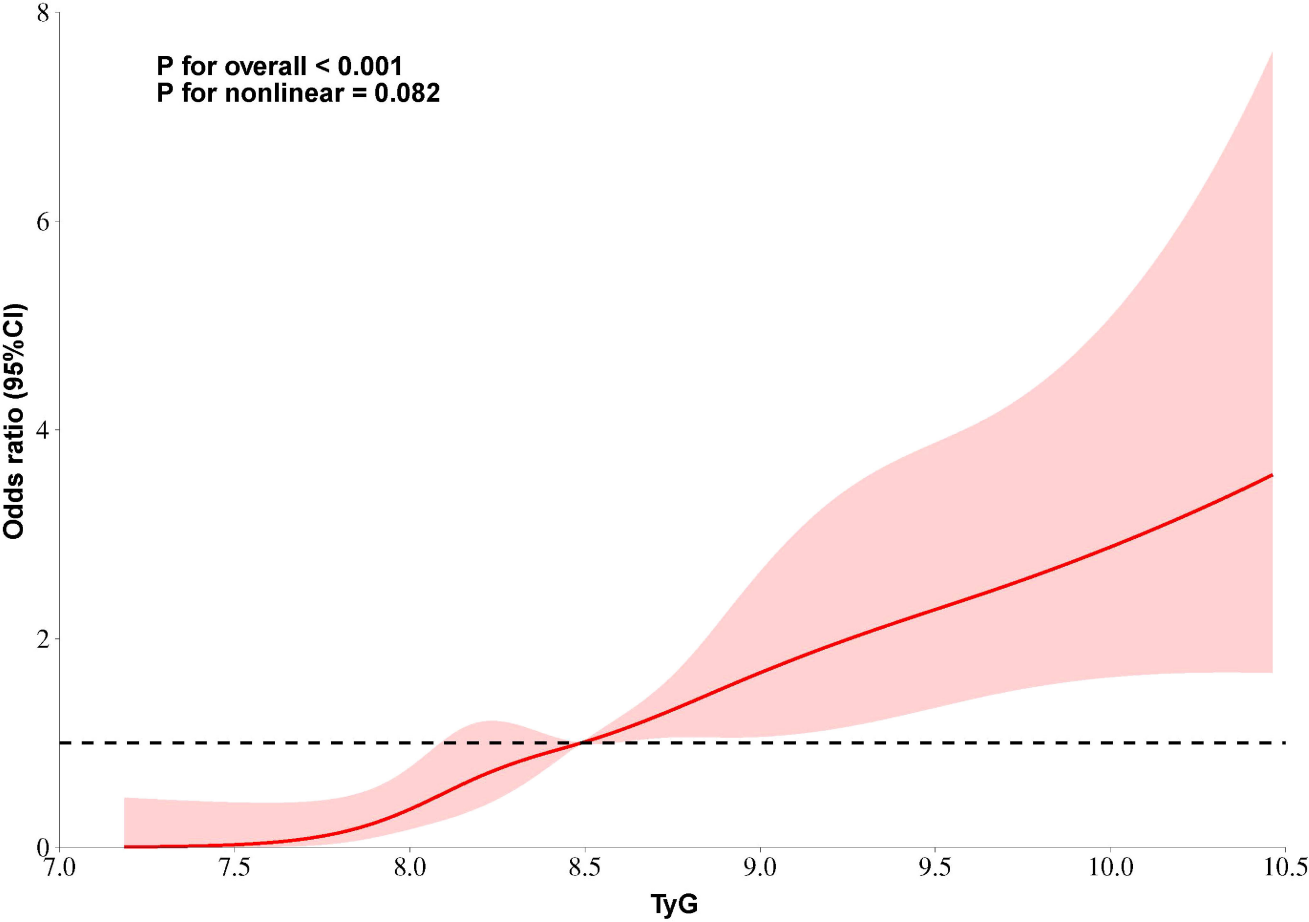
Curvilinear relationship between TyG index and risk of severe tubular atrophy/interstitial fibrosis. The solid line represents the estimated odds ratio (OR), and the shaded area indicates the 95% confidence interval. The P-value for nonlinearity was 0.082, suggesting a linear association between the TyG index and severe tubular atrophy/interstitial fibrosis.

Restricted cubic splines analysis revealed a linear association between the TyG index and T2 lesions (P for non-linearity=0.082), indicating a trend toward a positive correlation between higher TyG values and increased risk of developing severe renal tubular atrophy and interstitial fibrosis (**Figure 3**).

### 3.5 TyG Index as an independent risk factor for the incidence of severe tubular atrophy/interstitial fibrosis in IgAN

Based on the TyG index cut-off value, all individuals were classified into two groups: a high TyG group (TyG>8.69) and a low TyG group (TyG ≤ 8.69). To minimize potential confounding due to baseline imbalances in clinical and pathological characteristics between the two groups, 1:1 propensity score matching was performed using the greedy matching algorithm, resulting in a matched cohort of 425 patients in the low TyG group and 425 patients in the high TyG group. As shown in **Table 3**, in the matched cohort, significant differences between the two groups were observed only in BMI, Scr, 24-hour urine protein and eGFR (P <0.001). Regarding pathological features assessed by the MEST-C score, a significant intergroup difference was found solely in the proportion of tubular atrophy/interstitial fibrosis (T lesions) (P < 0.001).

**Table 3:** Baseline characteristics of the study cohort before and after propensity score matching by the TyG index cut-off.

Variables	Total	Before matching			After matching		
		Low TyG group (≤8.69)	High TyG group(>8.69)	P value	Low TyG group (≤8.69)	High TyG group(>8.69)	P value
Participants, (n)	1791	1169	682		425	425	
Age, years	43(36-53)	40(33-49)	47(39-56)	**<0.001**	47(37-56)	45(38-54)	0.593
Male, n(%)	760(42.4)	394(35.5)	366(53.7)	**<0.001**	209(49.2)	217(51.2)	0.583
BMI, (kg/m2)	23.05(20.85-25.35)	22.04(19.96-24.14)	24.76(22.76-26.81)	**<0.001**	23.24(21.26-25.00)	24.54(22.49-26.45)	**<0.001**
Hypertension, n(%)	195(10.9)	120(10.8)	75(10.9)	0.907	52(12.4)	47(11.6)	0.593
Smoking, n(%)	172(9.6)	75(6.8)	97(14.2)	**<0.001**	46(10.8)	57(13.4)	0.248
HB, g/L	126(113-139)	126(114-138)	131(114-144)	**<0.001**	128(116-139)	128(113-143)	0.968
Albumin, g/L	38.5(35.8-41.0)	38.5(35.8-41.1)	38.3(34.8-40.8)	0.110	38.2(35.6-40.6)	38.5(35.3-41.0)	0.682
UA, umol/L	365.0(300.0-437.0)	335.0(278.0-405.0)	415.5(354.0-492.0)	**<0.001**	386(326-450)	400(336-461)	0.108
Scr, umol/L	80(62-109)	75(60-98)	97(73-142)	**<0.001**	86(66-111)	94(71-135)	**<0.001**
24h Upro, g/L	0.91(0.45-1.80)	0.71(0.38-1.34)	1.48(0.72-2.69)	**<0.001**	0.96(0.49-1.83)	1.17(0.62-2.46)	**<0.001**
eGFR, ml/min/1.73m^2^	85.20(61.00-106.60)	91.60(70.40-110.00)	67.70(45.50-92.70)	**<0.001**	80.80(60.80-100.20)	72.85(48.60-97.33)	**<0.001**
Serum IgG, mg/dl	1080(909-1250)	1125(952-1280)	1060(904-1230)	**0.002**	1070(877-1260)	1070(907-1240)	0.880
Serum IgA, mg/dl	295(233-362)	289(230-354)	308(248-387)	**<0.001**	309(243-373)	300(244-370)	0.762
Serum IgM, mg/dl	104(74-139)	104(74-138)	95(67-130)	**0.030**	99(71-133)	100(71-134)	0.836
Serum C3, mg/dl	97(86-110)	91(81-103)	105(95-116)	**<0.001**	100(90-112)	101(91-111)	0.972
Serum C4, mg/dl	23(19-28)	22(18-26)	26(22-30)	**<0.001**	25(21-28)	25(21-29)	0.855
Mesangial cellularity, n(%)							
M0	22(1.3)	11(0.9)	11(1.6)	0.247	5(1.2)	6(1.4)	0.762
M1	1769(98.7)	1098(99.1)	671(98.4)		420(98.8)	419(98.6)	
Endocapillary hypercellularity, n(%)							
E0	1232(68.8)	755(68.1)	477(69.9)	0.409	293(68.9)	285(67.1)	0.556
E1	559(31.2)	354(31.9)	205(30.1)		132(31.1)	140(32.9)	
Segmental sclerosis, n(%)							
S0	345(19.3)	208(18.8)	137(20.1)	0.488	79(18.6)	75(17.7)	0.722
S1	1446(80.7)	901(81.2)	545(79.9)		346(81.4)	350(82.3)	
Tubular atrophy/interstitial fibrosis, n(%)							
T0	1255(70.1)	865(78.0)	390(57.2)	**<0.001**	293(68.9)	247(58.2)	**<0.001**
T1	414(23.1)	202(18.2)	212(31.1)		106(24.9)	123(28.9)	
T2	122(6.8)	42(3.8)	80(11.7)		26(6.2)	55(12.9)	
Crescents, n(%)							
C0	642(35.8)	387(34.9)	255(37.4)	0.141	149(35.1)	145(34.1)	0.911
C1	1004(56.1)	640(57.7)	364(53.4)		241(56.7)	247(58.1)	
C2	145(8.1)	82(7.4)	63(9.2)		35(8.2)	33(7.8)	
Steroid with/without immunosuppressants treatment, n(%)	512(28.6)	309(27.9)	203(29.7)	0.387	121(28.5)	121(28.5)	1.000
Composite event, n(%)	130(7.3)	54(4.9)	76(11.1)	**<0.001**	31(7.3)	51(12.0)	**0.020**

Continuous variables are expressed as median (interquartile range); Categorical variables are expressed as frequency (%); Bold values was that the differences were significant (P<0.05).

BMI, body mass index; Hb, hemoglobin; TC, total cholesterol; TG, triglyceride; LDL, low-density lipoprotein; FBG, fasting blood glucose; UA, uric acid; Scr, serum creatinine; 24h Upro, 24-hour urine protein; eGFR, estimated glomerular filtration rate.

Univariate logistic regression identified a TyG index >8.69 as a significant predictor of severe tubular atrophy/interstitial fibrosis (T2 lesions) in IgAN. Compared to patients with TyG ≤8.69, those with TyG >8.69 exhibited a 2.376-fold increase in the odds of T2 lesions (OR = 3.376,95%CI:2.294-4.969,p<0.001).Other factors significantly associated with T2 included gender, smoking status, hemoglobin, albumin, uric acid>420 μmol/L, 24-hour urine protein >3.5 g, eGFR ≤30 mL/min/1.73 m², serum IgG, and the presence of segmental sclerosis (S1) or cellular/fibrocellular crescents (C2).After adjusting for these covariates, multivariate logistic regression confirmed that the TyG index remained an independent risk factor for T2 lesions (OR = 2.365, 95% CI: 1.104–5.062, p=0.027), indicating a 1.365-fold increase in odds compared to the reference group. We also found that gender, hemoglobin, uric acid > 420 μmol/L, eGFR ≤ 30 mL/min/1.73 m², Serum C4,and the presence of segmental sclerosis (S1) were independent factors associated with severe tubular atrophy/interstitial fibrosis in IgAN patients, as shown in **Table 4**.

**Table 4:** Factors associated with severe tubular atrophy/interstitial fibrosis in 1791 IgAN patients (logistic regression analysis).

Variables	Univariate		Multivariate	
	OR(95%CI)	P value	OR(95%CI)	P value
TyG>8.69	3.376(2.294-4.969)	**<0.001**	2.365(1.104-5.062)	**0.027**
Age, years	1.012(0.996-1.027)	0.139	0.970(0.941-1.001)	0.056
Male, n(%)	2.292(1.572-3.343)	**<0.001**	3.129(1.243-7.878)	**0.015**
BMI, (kg/m^2^)	1.000(0.987-1.012)	0.981	0.980(0.907-1.059)	0.611
Hypertension, n(%)	1.066(0.598-1.899)	0.829	2.274(0.752-6.881)	0.146
Smoking, n(%)	2.517(1.562-4.056)	**<0.001**	1.869(0.750-4.659)	0.179
HB, g/L	0.955(0.945-0.965)	**<0.001**	0.952(0.931-0.974)	**<0.001**
Albumin, g/L	0.893(0.867-0.919)	**<0.001**	0.975(0.895-1.061)	0.553
UA>420, umol/L	6.990(4.638-10.535)	**<0.001**	4.869(2.249-10.586)	**<0.001**
24h Upro>3.5, g/L	9.701(6.466-14.555)	**<0.001**	2.372(0.905-6.217)	0.079
eGFR ≤ 30, ml/min/1.73m^2^	26.835(17.602-40.910)	**<0.001**	20.255(8.726-47.018)	**<0.001**
Serum IgG, mg/dl	0.999(0.998-1.000)	**<0.001**	1.000(0.999-1.001)	0.832
Serum IgA, mg/dl	1.001(0.999-1.003)	0.291	1.001(0.997-1.004)	0.783
Serum IgM, mg/dl	0.998(0.995-1.002)	0.373	1.007(1.000-1.014)	0.063
Serum C3, mg/dl	0.999(0.989-1.009)	0.846	0.993(0.972-1.015)	0.545
Serum C4, mg/dl	1.025(1.004-1.046)	**0.017**	1.002(1.001-1.044)	**0.041**
E1(VS E0)	1.216(0.827-1.789)	0.320	0.944(0.446-1.995)	0.879
S1(VS S0)	5.986(2.407-14.768)	**<0.001**	44.435(4.290-46.238)	**0.001**
C1(VS C0)	0.818(0.545-1.228)	0.333	1.090(0.479-2.478)	0.838
C2(VS C0)	2.302(1.322-4.008)	**0.003**	0.357(0.091-1.401)	0.140

Bold values was that the differences were significant (P<0.05).

BMI, body mass index; Hb, hemoglobin; UA, uric acid;Scr,serum creatinine; 24h Upro, 24-hour urine protein; eGFR, estimated glomerular filtration rate; M, mesangial proliferation; E, endocapillary proliferation; S, segmental glomerulosclerosis; T, tubular atrophy or interstitial fibrosis; C, crescents; OR, Odds ratios; CI, confidence intervals.

### 3.6 TyG index as an independent risk factor for the progression of IgAN to ESRD

**Figure 4:**
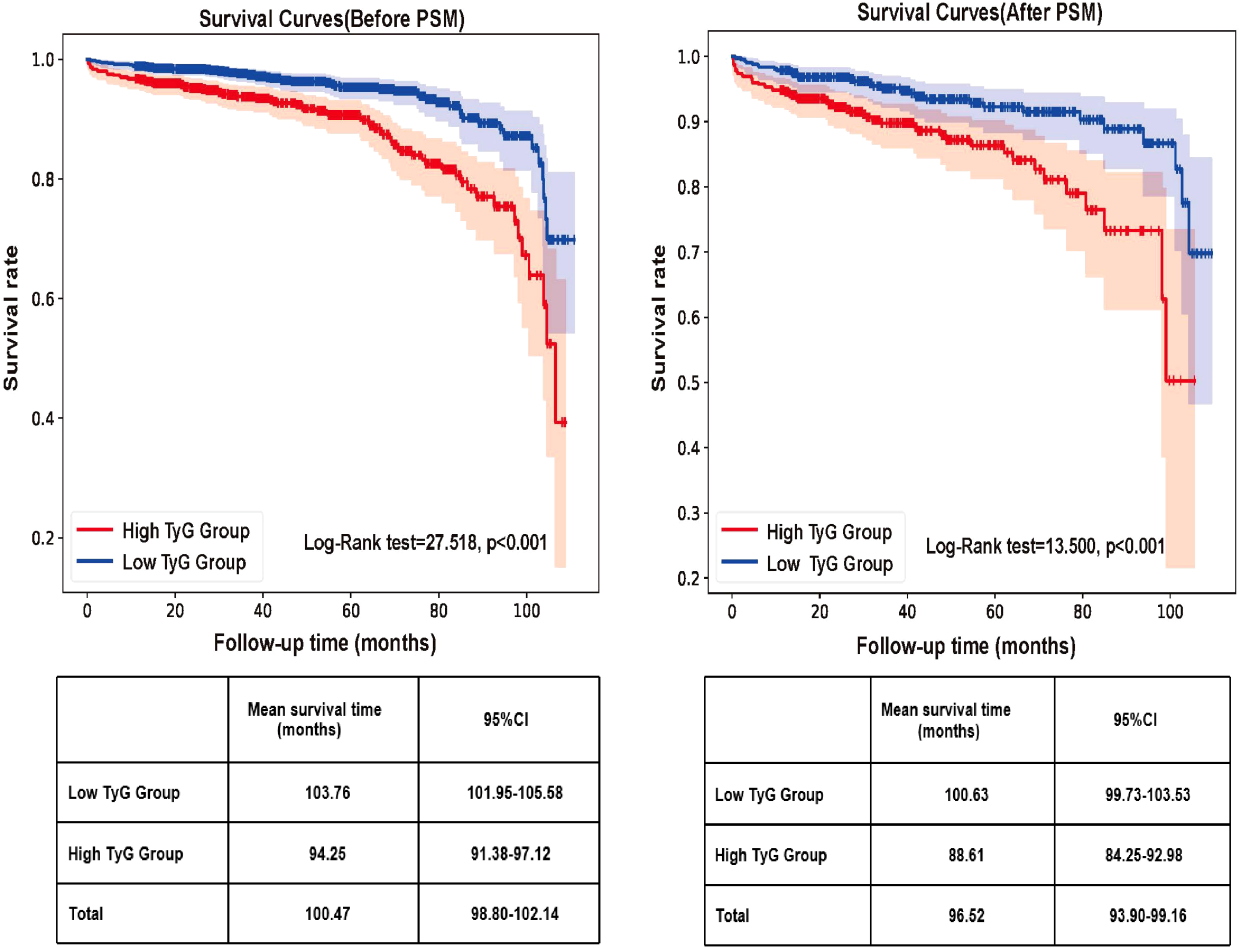
Kaplan–Meier analysis for renal survival between the high TyG group and the low TyG group in the unmatched and matched cohorts. Log-rank tests were used to compare survival distributions. After matching, the high-TyG group showed significantly worse renal survival (log-rank χ² = 13.59, P < 0.001).

Kaplan-Meier analysis demonstrated a significantly higher risk of kidney failure among patients in the high TyG group (log-rank χ² = 13.500, P < 0.001), with the low TyG group exhibiting a substantially longer mean renal survival time in the matched cohort (**Figure 4**). Using a predefined cutoff, Cox regression analyses were performed to evaluate the association between TyG index and adverse renal outcomes. In the unadjusted model, a high TyG level was associated with a markedly increased risk (HR = 2.470;95% CI: 1.741-3.502; P < 0.001). To control for potential confounders, three multivariate models were sequentially constructed with incremental adjustments for clinical, pathological, and treatment-related covariates. After adjustment for clinical parameters (including age, sex, hemoglobin, albumin, 24-hour urine protein, and eGFR), the TyG index remained significantly associated with disease progression (adjusted HR = 1.846;95%CI: 1.238-2.753; P = 0.003). This association persisted after further adjustment for Oxford MEST-C pathological scores (adjusted HR = 1.567; 95%CI: 1.051-2.337; P = 0.027) and additionally for treatment-related variables (adjusted HR = 1.561; 95% CI: 1.046–2.330; P = 0.029), as detailed in **Table 5**. These results confirm that an elevated TyG index (TyG >8.69) is an independent predictor of renal outcome in patients with IgAN.

**Table 5:** Multivariate Cox regression analysis of the association between the TyG index cut-off and renal outcomes.

	Low TyG group (≤8.69)	High TyG group (>8.69)	P value
Crude Model	1.00(reference)	2.470 (1.741-3.502)	**<0.001**
Model 1	1.00(reference)	1.846 (1.238-2.753)	**0.003**
Model 2	1.00(reference)	1.567 (1.051-2.337)	**0.027**
Model3	1.00(reference)	1.561 (1.046-2.330)	**0.029**

Model 1: was adjusted for age, gender+clinic factors (hemoglobin, albumin, 24h Upro, eGFR); Model 2: was adjusted for Model 1+Oxford (MEST-C). Model 3: was adjusted for Model 2 + treatment. eGFR was transformed into a binary variable with a cutoff of 30. Tubulointerstitial atrophy/interstitial fibrosis (T) was transformed into a binary variable of T0 + T1 and T2. Crescent (C) was transformed into a binary variable of C0 + C1 and C2. CI, confidence intervals; HR, hazard ratios. Bold values was that the differences were significant.

### 3.7 Multivariate ROC analysis of the TyG-integrated IIgA-PRT model

**Figure 5:**
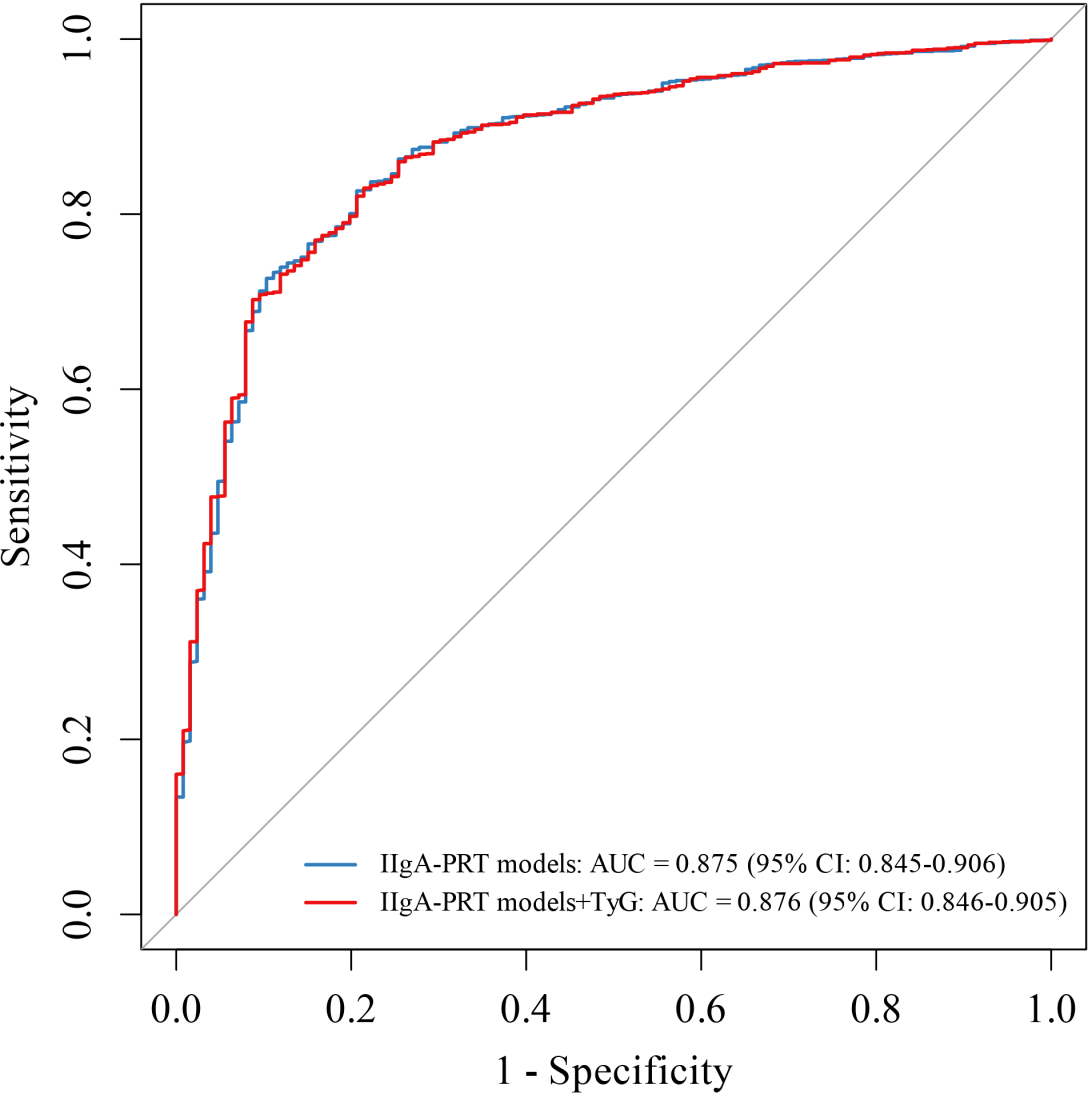
The ROC of IIgAN-PRT models with and without TyG. The integrated model (TyG + IIgAN-PRT) yielded an AUC of 0.876, slightly higher than the original IIgAN-PRT model (AUC = 0.875). The curves illustrate the incremental discriminative ability of adding the TyG index to the existing prognostic model. AUC, the area under the receiver operating characteristic curve.

An enhanced version of the IIgA-PRT model was developed by integrating the TyG index with established predictors through multivariable logistic regression. Receiver operating characteristic (ROC) curve analysis indicated that the TyG-integrated model yielded a slightly improved area under the curve (AUC = 0.876) compared to the original IIgA-PRT model (AUC = 0.875), as shown in **Figure 5**.

## 4 Discussion

Currently, the diagnosis of IgAN continues to rely heavily on renal biopsy. Within the Oxford Classification system, tubular atrophy/interstitial fibrosis represents a key histologic lesion and an independent predictor of progression to end-stage renal disease (ESRD) (15). Consequently, identifying non-invasive markers capable of reflecting this pivotal pathological process represents a significant unmet clinical need. Although several clinical parameters, such as hemoglobin (16), homocysteine (17), inflammatory indices (18), uric acid (19), and triglycerides (20), have been associated with fibrotic progression, a biomarker that integrates the core metabolic and immunologic disturbances underlying IgAN progression remains lacking. The TyG index is a well-established surrogate for insulin resistance and has demonstrated prognostic value in cardiovascular and renal diseases, including IgAN (14). However, its direct and specific association with the severity of the defining histopathological lesion—tubulointerstitial fibrosis, and its potential role as an integrative immunometabolic mediator in IgAN pathogenesis has not yet been elucidated.

This study presents novel and significant evidence that enhances the established prognostic value of the TyG index. In a large, well-characterized cohort of 1,791 biopsy-proven IgAN patients, we demonstrate for the first time that a higher TyG index is independently associated with severe tubular atrophy/interstitial fibrosis (T2 lesions), even after rigorous adjustments for key clinical, laboratory, and pathological confounders (OR = 2.365, 95% CI: 1.104–5.062, p = 0.027). This finding directly addresses a critical knowledge gap regarding the relationship between metabolic dysregulation and specific histological injury in IgAN. Clinically, patients in the high-TyG group exhibited a more advanced disease phenotype, characterized by lower eGFR, higher serum creatinine, greater proteinuria, and notably elevated levels of serum IgA, C3, and C4. This observation suggests that the TyG index may reflect not only insulin resistance but also a state of complement system activation and heightened humoral immunity, indicating a novel immunometabolic nexus in IgAN. Kaplan-Meier analysis revealed a significant survival disadvantage in the high-TyG group (log-rank test, P < 0.001). However, it is important to note that residual imbalances in baseline renal function persisted even after propensity score matching. Therefore, more robust evidence supporting the independent prognostic value of the TyG index comes from the multivariable Cox regression models. These models sequentially adjusted for key clinical variables, Oxford MEST-C scores, and treatment regimens, and consistently identified a high TyG index as an independent risk factor for adverse renal outcomes (**Table 5**), thereby confirming its robust prognostic utility. Collectively, our findings reposition the TyG index from a general metabolic risk marker to a specific, non-invasive indicator of high-risk tubulointerstitial pathology and a potential integrative biomarker that captures both metabolic and immunological dimensions in IgAN.

The TyG index, a simple and reliable marker of IR, is derived exclusively from two readily available metabolic parameters:TG and FBG levels, the underlying mechanisms by which an elevated TyG index contributes to severe tubular atrophy/interstitial fibrosis warrant a comprehensive analysis, focusing on the respective roles of its two components. Liu et al. (20) identified hypertriglyceridemia as an independent risk factor for tubular atrophy/interstitial fibrosis in a cohort of patients with chronic kidney disease (CKD).Hypertriglyceridemia promotes abnormal lipid accumulation in renal cells, thereby triggering a cascade of key pathological processes, including oxidative stress, inflammatory cell infiltration, and fibroblast activation, which collectively drive renal tubular injury and the progression of interstitial fibrosis (21).A large-scale study involving 698 patients with IgAN demonstrated a positive correlation between FBG levels and 24-hour urinary protein excretion and inverse association with eGFR (22). Elevated FBG promotes renal oxidative stress through pathways involving advanced glycation end products (AGEs) and protein kinase C (PKC), which in turn stimulate processes like smooth muscle cell proliferation, collagen cross-linking, and deposition, ultimately contributing to renal injury (23). Emerging evidence indicates that certain insulin resistance-related signaling pathways may directly contribute to renal fibrogenesis, extending beyond classical metabolic insults. Du H et al. (24) demonstrated that SOX4, a transcription factor involved in regulating the insulin signaling pathway, promotes the dedifferentiation of tubular epithelial cells and the activation of fibroblasts by modulating Smad3 phosphorylation, thereby facilitating the progression of renal fibrosis. Similarly, signal transducer and activator of transcription 6 (STAT6) is activated in renal tubular cells under metabolic stress and has been implicated in macrophage polarization and fibroblast activation (25). The cGAS-STING pathway, a crucial mediator of the innate immune response to cytosolic DNA, has recently been linked to metabolic inflammation and renal fibrosis; its activation, induced by metabolic dysregulation, contributes to the formation of a pro-fibrotic microenvironment (26). Phosphatase and tensin homolog (PTEN), a key regulator of the PI3K/Akt signaling pathway downstream of insulin signaling, exacerbates kidney injury under deficient conditions by promoting fibroblast recruitment and activation (27). Although the precise interactions among these pathways and the pathogenesis of IgAN warrant further investigation, their well-documented roles in insulin resistance and renal fibrosis suggest plausible mechanistic links.

Furthermore, our study identified a significant association between the TyG index and serum levels of IgA, C3, and C4 in patients with IgAN. This finding is particularly relevant given that IgAN is an immune-mediated chronic inflammatory disease in which complement system activation plays a pivotal role in disease progression. Prior clinical evidence has demonstrated that serum C3 and C4 levels reflect the degree of complement activation and are associated with both the severity of renal inflammation and adverse renal outcomes in IgAN (28). Beyond its established association with metabolic dysregulation, the TyG index may also serve as an indicator of complement system activation in IgAN, suggesting a novel immunometabolic nexus in the pathogenesis of this disease. The underlying mechanisms require further investigation.

In addition to the TyG index, our study found that male sex, hemoglobin, hyperuricemia (UA > 420 μmol/L), reduced eGFR (<30 ml/min/1.73m²), and Serum C4 are also significantly and independently associated with severe renal tubular atrophy/interstitial fibrosis. Previous studies have consistently established that hemoglobin (16), uric acid (19), and eGFR (29) are closely associated with tubular atrophy/interstitial fibrosis in IgAN, findings consistent with our own. Male sex is an established independent risk factor for adverse renal outcomes in IgAN (1), a disparity often driven by the more severe clinical and pathological presentations observed in male patients, which may stem from underlying differences in sex hormone profiles and immune response dynamics. Bi et al. (30) found that elevated C4 levels were significantly associated with lower eGFR, segmental glomerulosclerosis, and tubular atrophy/interstitial fibrosis, and served as a useful predictor for disease progression in IgAN. Serum C4 serves as a key biomarker of complement activation, dysregulated activation promotes the generation of anaphylatoxins and membrane attack complexes, which trigger mesangial cells to release inflammatory mediators and overproduce extracellular matrix, further amplifying renal injury (28).

Furthermore, our study demonstrates that the TyG index possesses significant prognostic value for long-term renal outcomes in IgAN. Kaplan-Meier analysis revealed substantially lower renal survival in patients with high TyG levels. Critically, in multivariate Cox regression models, a TyG index >8.69 remained an independent predictor of progression to ESRD after sequential adjustment for clinical parameters, MEST-C scores, and immunosuppressive therapy, a finding consistent with the report by Qin A et al. (14).The inclusion of the TyG index in the IIgA-PRT model resulted in a marginal improvement in the AUC, increasing from 0.875 to 0.876. While this suggests a modest incremental value in statistical discrimination, the clinical significance of such a small enhancement remains uncertain and is likely negligible. Our primary finding underscores the TyG index as an independent risk factor for adverse renal outcomes, rather than advocating for its routine incorporation into established clinical prediction models. Our results demonstrate that the TyG index provides incremental prognostic value beyond conventional risk assessment tools. The observed association, although attenuated, remained statistically significant after adjustment for the MEST-C score, suggesting that chronic histopathological lesions such as tubular atrophy/interstitial fibrosis partially mediate the pathway linking the TyG index to poor renal prognosis in IgAN patients.

While the homeostasis model assessment of insulin resistance (HOMA-IR) is regarded as the gold standard for evaluating IR (31), its clinical utility is limited by operational complexity, high cost, and susceptibility to measurement inaccuracies in insulin assays (32). In contrast, the TyG index—derived solely from routine fasting blood measurements—has emerged as a well-validated, cost-effective, and readily accessible surrogate marker of IR. Our study further establishes the clinical relevance of the TyG index by demonstrating its independent association with tubulointerstitial damage and renal prognosis in patients with IgAN, supporting its use in risk stratification. These findings suggest that IgAN patients with elevated TyG levels may benefit from intensified monitoring and personalized management. Importantly, given that IR represents a modifiable pathophysiological driver, our results raise the possibility that interventions designed to improve insulin sensitivity—such as lifestyle modifications or metabolically active agents including SGLT2 inhibitors or metformin—may attenuate the progression of chronic kidney damage in this population. This compelling hypothesis merits further investigation in prospective interventional trials.

In this study, patients were divided at the optimal TyG index cut-off of 8.69, which had an AUC of 0.699 for predicting severe tubular atrophy and interstitial fibrosis. A TyG index >8.69 was linked to higher odds of these lesions in IgAN and increased risk of adverse renal outcomes. While the AUC indicates moderate accuracy, it supports the TyG index as a non-invasive biomarker and justifies further research. Incorporating the TyG index into a multi-parameter model enhanced predictive performance, yielding a modest yet significant improvement in discrimination and affirming its status as an independent metabolic risk factor. Although the model demonstrated strong performance with routine clinical variables, external validation across diverse populations is essential prior to clinical implementation. Future research should assess the cost-effectiveness and practical applicability of this improved TyG-based model.

Nevertheless, this study has several limitations. First, as a single-center, retrospective cohort study conducted within a Chinese population and characterized by a relatively short follow-up period, it is subject to inherent selection bias, which may limit the generalizability of our findings. Second, while the sample size was not predetermined, all eligible patients during the study period were included, resulting in a substantial sample (n=1,791) that ensures statistical reliability. However, prospective studies would benefit from formal power calculations to enhance methodological rigor. Third, the impact of longitudinal changes in the TyG index on clinical outcomes remains unexamined. The TyG index was assessed only at baseline, and the association between changes in the TyG index and key clinical outcomes, such as eGFR decline and proteinuria, was not evaluated. Consequently, it is unclear whether lowering the TyG index improves kidney outcomes, which limits its current value as a therapeutic target. Fourth, although propensity score matching was applied to minimize potential confounding, residual imbalances in key baseline characteristics, specifically BMI, Scr, 24h Upro, and eGFR, remained between the high- and low-TyG groups in the matched cohort (**Table 3**). These differences suggest that individuals in the high TyG group had a more advanced disease phenotype at baseline. Consequently, while the Kaplan-Meier analysis (**Figure 4**) shows a visually apparent survival difference, this disparity may be partially attributable to residual confounding. Therefore, greater weight should be given to the results of the multivariable Cox regression models (**Table 5**), which adjusted for these and other important clinical, pathological, and treatment-related covariates, and consistently identified the TyG index as an independent predictor of adverse renal outcomes. Fifth, the retrospective nature of this study precludes definitive causal conclusions regarding the relationship between the TyG index and severe tubular atrophy/interstitial fibrosis. Most importantly, the optimal TyG index cut-off of 8.69 was established through internal validation within a single-center cohort; thus, its generalizability and clinical applicability remain uncertain without external validation from an independent, multi-center population. Therefore, large-scale, multicenter, prospective studies with extended follow-up are warranted to validate and expand upon these findings.

## 5 Conclusion

In conclusion, the TyG index is a novel and independent biomarker that is significantly associated with severe tubular atrophy/interstitial fibrosis and predicts adverse renal outcomes in patients with IgAN. For IgAN patients presenting with a TyG index >8.69 at the time of renal biopsy, more intensive monitoring and management should be considered.

## Data Availability

The original contributions presented in the study are included in the article/supplementary material. Further inquiries can be directed to the corresponding author.

## References

[1] Pitcher D Braddon F Hendry B Mercer A Osmaston K Saleem MA . Long-term outcomes in igA nephropathy. Clin J Am Soc Nephrol. (2023) 18:727–38. doi: 10.2215/CJN.0000000000000135, PMID: 37055195 PMC10278810

[2] Shen X Chen P Liu M Liu L Shi S Zhou X . Long-term outcomes of IgA nephropathy in China. Nephrol Dial Transplant. (2025) 40:1137–46. doi: 10.1093/ndt/gfae252, PMID: 39557651

[3] Stamellou E Seikrit C Tang SCW Boor P Tesař V Floege J . IgA nephropathy. Nat Rev Dis Primers. (2023) 9:67. doi: 10.1038/s41572-023-00476-9, PMID: 38036542

[4] Saleem N Nasir H Anwar F Aziz M Khurshid K Bashir S . To evaluate the utility of Oxford classification in predicting renal outcome in IgA nephropathy patients. Int Urol Nephrol. (2024) 56:345–53. doi: 10.1007/s11255-023-03685-z, PMID: 37378850

[5] Howie AJ Lalayiannis AD . Systematic review of the oxford classification of igA nephropathy: reproducibility and prognostic value. Kidney360. (2023) 4:1103–11. doi: 10.34067/KID.0000000000000195, PMID: 37357346 PMC10476683

[6] Ramdas Nayak VK Satheesh P Shenoy MT Kalra S . Triglyceride Glucose (TyG) Index: A surrogate biomarker of insulin resistance. J Pak Med Assoc. (2022) 72:986–8. doi: 10.47391/JPMA.22-63, PMID: 35713073

[7] Wei X Min Y Song G Ye X Liu L . Association between triglyceride-glucose related indices with the all-cause and cause-specific mortality among the population with metabolic syndrome. Cardiovasc Diabetol. (2024) 23:134. doi: 10.1186/s12933-024-02215-0, PMID: 38658993 PMC11044377

[8] da Silva A Caldas APS Rocha DMUP Bressan J . Triglyceride-glucose index predicts independently type 2 diabetes mellitus risk: A systematic review and meta-analysis of cohort studies. Prim Care Diabetes. (2020) 14:584–93. doi: 10.1016/j.pcd.2020.09.001, PMID: 32928692

[9] Yang Y Huang X Wang Y Leng L Xu J Feng L . The impact of triglyceride-glucose index on ischemic stroke: a systematic review and meta-analysis. Cardiovasc Diabetol. (2023) 22:2. doi: 10.1186/s12933-022-01732-0, PMID: 36609319 PMC9825038

[10] Khalaji A Behnoush AH Khanmohammadi S Ghanbari Mardasi K Sharifkashani S Sahebkar A . Triglyceride-glucose index and heart failure: a systematic review and meta-analysis. Cardiovasc Diabetol. (2023) 22:244. doi: 10.1186/s12933-023-01973-7, PMID: 37679763 PMC10486123

[11] Xin F He S Zhou Y Jia X Zhao Y Zhao H . The triglyceride glucose index trajectory is associated with hypertension: a retrospective longitudinal cohort study. Cardiovasc Diabetol. (2023) 22:347. doi: 10.1186/s12933-023-02087-w, PMID: 38102704 PMC10725029

[12] Siddiqui K Nawaz SS George TP David SK Alfadda AA Rafiullah M . Association of triglyceride-glucose index with diabetic kidney disease in patients with type 2 diabetes. J Diabetes Metab Disord. (2025) 24:171. doi: 10.1007/s40200-025-01680-y, PMID: 40678701 PMC12263496

[13] Kunutsor SK Seidu S Kurl S Laukkanen JA . Baseline and usual triglyceride-glucose index and the risk of chronic kidney disease: a prospective cohort study. Geroscience. (2024) 46:3035–46. doi: 10.1007/s11357-023-01044-5, PMID: 38180700 PMC11009217

[14] Qin A Tan J Wang S Dong L Jiang Z Yang D . Triglyceride-glucose index may predict renal survival in patients with igA nephropathy. J Clin Med. (2022) 11:5176. doi: 10.3390/jcm11175176, PMID: 36079108 PMC9456599

[15] Sugiura N Moriyama T Miyabe Y Karasawa K Nitta K . Severity of arterial and/or arteriolar sclerosis in IgA nephropathy and the effects of renin-angiotensin system inhibitors on its prognosis. J Pathol Clin Res. (2021) 7:616–23. doi: 10.1002/cjp2.234, PMID: 34185389 PMC8503890

[16] Chen X Hu H Wan Q . The relationship between hemoglobin and renal tubular atrophy/interstitial fibrosis in patients with IgA nephropathy [in Chinese. Chin J Nephrol. (2020) 36:106–14. doi: 10.3760/cma.j.issn.1001-7097.2020.02.007

[17] Li Z Han Q Ye H Li J Wei X Zhang R . Serum homocysteine is associated with tubular interstitial lesions at the early stage of IgA nephropathy. BMC Nephrol. (2022) 23:78. doi: 10.1186/s12882-021-02632-3, PMID: 35196994 PMC8867621

[18] Liang X Jiang X . The hemoglobin, albumin, lymphocyte, and platelet (HALP) score is associated with severe renal tubular atrophy/interstitial fibrosis in IgA nephropathy. Eur J Med Res. (2024) 29:542. doi: 10.1186/s40001-024-02148-0, PMID: 39533443 PMC11558843

[19] Fang B Zheng Y Wang Y Yu Y Dong X . Analysis of risk factors for tubular atrophy/interstitial fibrosis in primary membranous nephropathy. Med (Baltimore). (2025) 104:e43455. doi: 10.1097/MD.0000000000043455, PMID: 40725876 PMC12303468

[20] Liu B Zhao L Yang Q Zha D Si X . Hyperuricemia and hypertriglyceridemia indicate tubular atrophy/interstitial fibrosis in patients with IgA nephropathy and membranous nephropathy. Int Urol Nephrol. (2021) 53:2321–32. doi: 10.1007/s11255-021-02844-4, PMID: 33895976

[21] Du XG Ruan XZ . Lipid metabolism disorder and renal fibrosis. Adv Exp Med Biol. (2019) 1165:525–41. doi: 10.1007/978-981-13-8871-2_26, PMID: 31399983

[22] Ma H Lei C Zhao B Feng Z Ye L Wang X . The impact of metabolic component count on IgA nephropathy prognosis. Sci Rep. (2024) 14:30996. doi: 10.1038/s41598-024-81929-3, PMID: 39730781 PMC11680595

[23] Kitada M Kume S Imaizumi N Koya D . Resveratrol improves oxidative stress and protects against diabetic nephropathy through normalization of Mn-SOD dysfunction in AMPK/SIRT1-independent pathway. Diabetes. (2011) 60:634–43. doi: 10.2337/db10-0386, PMID: 21270273 PMC3028365

[24] Du H Jiao B Xing J Yang D Tran M Wang P . Critical role of transcription factor SOX4 in tubular epithelial cell dedifferentiation and fibroblast activation during kidney fibrosis. Kidney Int. (2025) 109:S0085–2538(25)00762-8. doi: 10.1016/j.kint.2025.08.030. Epub ahead of print, PMID: 41005570 PMC12548802

[25] Jiao B An C Du H Tran M Wang P Zhou D . STAT6 deficiency attenuates myeloid fibroblast activation and macrophage polarization in experimental folic acid nephropathy. Cells. (2021) 10:3057. doi: 10.3390/cells10113057, PMID: 34831280 PMC8623460

[26] Jiao B An C Du H Tran M Yang D Zhao Y . Genetic deficiency or pharmacological inhibition of cGAS-STING signalling suppresses kidney inflammation and fibrosis. Br J Pharmacol. (2025) 182:1741–62. doi: 10.1111/bph.17412, PMID: 39833988

[27] An C Jiao B Du H Tran M Zhou D Wang Y . Myeloid PTEN deficiency aggravates renal inflammation and fibrosis in angiotensin II-induced hypertension. J Cell Physiol. (2022) 237:983–91. doi: 10.1002/jcp.30574, PMID: 34515350 PMC8810675

[28] Tringali E Vetrano D Tondolo F Maritati F Fabbrizio B Pasquinelli G . Role of serum complement C3 and C4 on kidney outcomes in IgA nephropathy. Sci Rep. (2024) 14:16224. doi: 10.1038/s41598-024-65857-w, PMID: 39003309 PMC11246413

[29] Gan Y Cai Y Li J Wu J Zhang R Han Q . Development and validation of a diagnostic nomogram to evaluate tubular atrophy/interstitial fibrosis of IgA nephropathy. Int J Med Sci. (2024) 21:674–80. doi: 10.7150/ijms.91804, PMID: 38464822 PMC10920839

[30] Bi TD Zheng JN Zhang JX Yang LS Liu N Yao L . Serum complement C4 is an important prognostic factor for IgA nephropathy: a retrospective study. BMC Nephrol. (2019) 20:244. doi: 10.1186/s12882-019-1420-0, PMID: 31272400 PMC6610919

[31] Matthews DR Hosker JP Rudenski AS Naylor BA Treacher DF Turner RC . Homeostasis model assessment: insulin resistance and beta-cell function from fasting plasma glucose and insulin concentrations in man. Diabetologia. (1985) 28:412–9. doi: 10.1007/BF00280883, PMID: 3899825

[32] Shi Y Hu L Li M Zhou W Wang T Zhu L . Association between the surrogate markers of insulin resistance and chronic kidney disease in chinese hypertensive patients. Front Med (Lausanne). (2022) 9:831648. doi: 10.3389/fmed.2022.831648, PMID: 35198578 PMC8859105

